# Safety and Efficacy of Midline vs Peripherally Inserted Central Catheters Among Adults Receiving IV Therapy: A Randomized Clinical Trial

**DOI:** 10.1001/jamanetworkopen.2023.55716

**Published:** 2024-02-13

**Authors:** Simon L. Thomsen, Rikke Boa, Lars Vinter-Jensen, Bodil S. Rasmussen

**Affiliations:** 1 Department of Anesthesia and Intensive Care, Aalborg University Hospital, Aalborg, Denmark; 2 Department of Gastroenterology, Centre for Nutrition and Intestinal Failure, Aalborg University Hospital, Aalborg, Denmark; 3 Department of Clinical Medicine, Aalborg University, Aalborg, Denmark

## Abstract

**Importance:**

Midline catheters (MCs) are widely used, but safety and efficacy compared with peripherally inserted central catheters (PICCs) has not been adequately evaluated.

**Objective:**

To compare the safety and efficacy of MCs with PICCs among adult patients with an anticipated intravenous therapy lasting from 5 to 28 days.

**Design, Setting, and Participants:**

This parallel, 2-group, open-label, randomized clinical trial (RCT) was conducted in Denmark from October 2018 to February 2022 at a single academic tertiary care center. Adult inpatients and outpatients were consecutively randomized.

**Intervention:**

Patients were randomized in a 1:1 ratio to either the MC group or the PICC control group.

**Main Outcomes and Measures:**

The primary outcome was catheter-related bloodstream infection (CRBSI), analyzed using the Fisher exact test. Secondary outcomes were symptomatic catheter-related thrombosis and catheter failure, including mechanical cause, phlebitis, infiltration, pain in relation to drug or fluid administration, and leaking from the puncture site. Incidence rate ratios (IRRs) were calculated to assess between-group failure rates over device dwell time using Poisson regression. An intention-to-treat analysis was performed.

**Results:**

A total of 304 patients (mean [SD] age, 64.6 [13.5] years; 130 [42.8%] female) were included in the analysis, and 152 patients were allocated to each catheter group. The incidence of CRBSI was low, with 0 in the MC group and 1 in the PICC control group (*P* > .99). The MC group had a higher catheter-related complication rate (20 [13.2%] vs 11 [7.2%]), and an IRR of 2.37 (95% CI, 1.12-5.02; *P* = .02) for complications compared with the PICC control group. In a post hoc analysis stratified by catheter dwell time, no significant difference in complication rate (IRR, 1.16; 95% CI, 0.50-2.68; *P* = .73) was found between the 2 groups for catheters used less than 16 days.

**Conclusions and Relevance:**

In this RCT with patients who received medium- to long-term intravenous therapy, the incidence of CRBSI was low, with no difference between MCs and PICCs. The use of MCs resulted in a higher incidence of catheter-related complications compared with use of PICCs. This finding should be balanced in the decision of type of catheter used at the individual patient level.

**Trial Registration:**

ClinicalTrials.gov Identifier: NCT04140916

## Key Points

**Question:**

Is the use of midline catheters (MCs) a safe and more efficacious alternative to peripherally inserted central catheters (PICCs) for adult patients receiving medium- to long-term intravenous therapy?

**Findings:**

In this randomized clinical trial of 304 patients, rates of catheter-related bloodstream infection did not differ between the catheter groups. The MC group had a statistically significantly higher catheter-related complication rate, with an incidence rate ratio of 2.37 compared with the PICC control group.

**Meaning:**

In this study, MCs and PICCs were both safe and efficacious, and despite the MC group having a higher complication rate, MCs could be an alternative to PICCs.

## Teaser

This randomized clinical trial compares the safety and efficacy of midline catheters vs peripherally inserted central catheters among adult patients with an anticipated intravenous (IV) therapy lasting from 5 to 28 days.

## Introduction

Intravenous (IV) accesses are the most common invasive procedures, involving approximately 80% of all hospital admissions, with an average of 2 devices per patient during hospitalization.^1^ The indications are numerous and include administration of IV medication, fluid, parenteral nutrition, and blood products.^2^ Peripheral IV catheters are frequently used, as they are easily inserted bedside and have a low risk of complications.^3^ However, the relatively short dwell time of peripheral IV catheters, lasting approximately 3 to 4 days, requires repetitive insertions to complete medium- and long-term IV therapies.^4^ In these situations, central venous catheters or peripherally inserted central catheters (PICCs) are recommended.^5,6^

An alternative type of peripherally inserted IV catheter is the midline catheter (MC), which typically range from 8 to 20 cm in length and are inserted into the major veins of the upper arm, with the tip positioned in or distal to the axillary vein.^7^ MCs were introduced in the 1950s, but hypersensitivity reactions to the catheter material led to decline in their use.^8^ Since then, the manufacturing materials as well as the techniques of insertion have been improved. Current guidelines recommend the use of MCs for medium duration of IV therapies and in patients with difficult peripheral IV access.^6^ Nevertheless, these recommendations are largely based on expert opinions due to sparse evidence.

One of the major serious complications associated with use of any type of IV catheter is catheter-related bloodstream infection (CRBSI). In a systematic review of prospective studies published between 1966 and 2005, an estimated CRBSI rate of 0.4% with the use of 514 MCs and 3.1% with the use of 3566 PICCs was reported.^9^ Regular use of MCs may decrease the overall incidence of CRBSI.^10^ However, when compared with PICCs, MCs have been shown to be associated with a higher incidence of complications.^11^ Therefore, to obtain more conclusive findings, further investigation in larger randomized clinical trials (RCTs) comparing the new generation of MCs with PICCs is needed.

The aim of this superiority trial was to compare the safety and efficacy of MCs with PICCs among adult patients requiring medium- to long-term IV therapy lasting from 5 to 28 days. We hypothesized that the group of patients with MCs would experience a lower incidence of CRBSIs compared with the control group of patients with PICCs.

## Methods

### Study Design

We performed a single-center open-label, 2-group, parallel-group RCT to compare catheter complications between the new generation MC Vygon Seldipur Smartmidline (Vygon) (MC group) with a Cook Turbo-Ject Power-Injectable PICC (Cook Medical) or a B. Braun Celsite PICC-Cel (B. Braun) (PICC control group). The study took place at a university hospital in Denmark covering a local region with approximately 600 000 residents. The study was approved by the North Denmark Region Committee on Health Research Ethics and was conducted in accordance with the Helsinki II Declaration. All patients who participated in the trial provided informed consent before inclusion. This study followed the Consolidated Standards of Reporting Trials (CONSORT) guideline for reporting RCTs. A full trial protocol is given in Supplement 1.

### Participants

We consecutively enrolled both inpatients and outpatients who met the inclusion criteria. Eligible patients were enrolled if they were age 18 years or older and had an indication for IV medicine or fluid therapy, including blood products, isotonic saline- or glucose-solutions, antibiotics (penicillins, cephalosporins, carbapenems, or fluoroquinolones), and chemotherapy registered for use in peripheral venous catheters, with an anticipated need for an IV access lasting from 5 to 28 days. All infusates were approved by the manufacturer for administration in a peripheral IV catheter. Exclusion criteria included pregnancy, infection or burns at both upper extremities, existing central venous catheter, informed consent not obtainable, or earlier randomization to the study.

### Blinding and Randomization

Nurses from the vascular access team randomized the patients in a 1:1 ratio to either the MC group or the PICC control group using the online randomization tool Research Electronic Data Capture (REDCap).^12^ The study participants, the clinical team, and the researchers were not blinded to the intervention, as the devices used were visibly different.

### Training and Education

Five specifically trained nurses in insertion of IV accesses and part of the vascular access team performed all insertions of MCs and PICCs. The indications were provided by the anesthesiologist in charge who also supervised the nurses. Prior to the study, nurses on the wards and in the home care institutions were trained with specific use and care procedures for both catheters. Written materials were present for all procedures.

### Procedures

All patients were admitted to the hospital at the time of catheter insertion. The patient was placed in a supine position, and ultrasonography was used to identify veins on the relevant upper extremity. Sterile techniques were used, including operator equipped sterile gown, mask, cap, and sterile gloves. The area was prepared with chlorhexidine-alcohol followed by adequate sterile draping. After cannulation, successful insertion was confirmed by aspirating blood from the catheter. Subsequently, the tip position of the PICC was verified by a chest radiograph at the end of the procedure.

Following insertion, the puncture sites were inspected daily through transparent semipermeable dressings, replaced at intervals of no more than 7 days. Needleless connectors were used and flushing with 10 to 20 mL of saline was performed after each use. In the PICC group, heparin was installed if the catheter remained unused for more than 24 hours. Tissue-type plasminogen activator was not used in cases of catheter clotting.

Consecutive registrations were made for the date of insertion, indication for use, specific type of IV catheter, number of skin punctures, time spent in minutes, accidental arterial puncture, bleeding complications, and tip placement on chest radiograph for the PICC control group. Additionally, electronic medical record data (Clinical Suite [DXC Technology]) were collected including age, sex, body mass index, history of myocardial infarction, congestive heart failure, peripheral vascular disease, cerebrovascular disease, dementia, chronic pulmonary disease, connective tissue disease, ulcer disease, liver disease, diabetes with or without end organ failure, hemiplegia, kidney disease, tumor without metastasis including leukemia and lymphoma, metastatic solid tumor, and AIDS.

Patients were followed up until 90 days after the completion of IV therapy or death. Date of therapy completion and examination of potential catheter-tip colonization were noted, and if the catheter failed or was removed before IV therapy completion, details were documented. All outcomes were systematically tracked in the patients’ electronic medical records. Data were stored using the REDCap electronic data capture tools hosted at Aalborg University Hospital, Denmark.

### Outcomes

The primary outcome was the incidence of CRBSI registered from insertion until removal of the catheter. CRBSI was defined as the presence of clinical signs of infection (ie, fever, chills, leukocytosis, or hypotension) and at least 1 positive blood culture obtained directly from the catheter or a peripheral vein in the absence of other apparent source for the infection except the catheter. Additionally, CRBSI was quantitatively defined as more than 1000 colony-forming units per catheter tip culture with the same organism (species and anti-biogram) isolated from the catheter and peripheral blood culture. Secondary outcomes were deep vein thrombosis (DVT) defined by the formation of 1 or more symptomatic (ie, pain or swelling) blood clots in a large vein verified by ultrasonography or a computed tomography scan, catheter failure of mechanical cause (accidentally removed, removed by coincidence, clotted, leakage, or other defects of the catheter), phlebitis, infiltration, pain in relation to drug or fluid administration, or leaking from the puncture site.

### Statistical Analysis

Based on data before study initiation, we assumed an incidence of CRBSI of 0% in the MC group and 5% in the PICC control group.^9,13^ With a significance level (α) of .05 and a power of 80% (β = 0.2), the sample size was calculated to be 304, with 152 patients in each group.

Data were analyzed by intention-to-treat. After a test for normal distribution, comparisons between the groups were made using 2-sample *t* test or Wilcoxon rank sum test for continuously measured variables and Fisher exact test for categorical variables.

We used Poisson regression with robust variance estimation to estimate the incidence rates and the incidence rate ratios (IRRs) for premature catheter removal and all-cause complication, with the total number of catheter-days as the denominator. Two-sided analyses were performed, and *P* < .05 was considered statistically significant. All statistical analyses were performed with Stata version 17.0 MP software (StataCorp).

## Results

**Figure 1.**
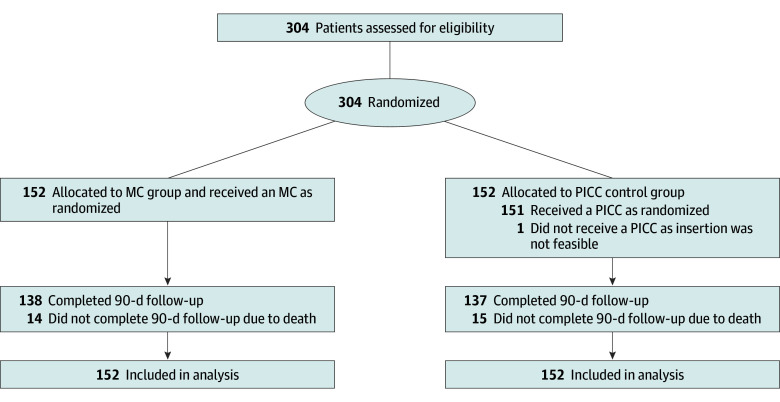
Flow Diagram for the Randomization and Analysis of Patients MC indicates midline catheter; PICC, peripherally inserted central catheter.

From October 2018 to February 2022, a total of 304 patients (mean [SD] age, 64.6 [13.5] years; age range, 21-89 years; 130 [42.8%] female) were randomized, of whom 152 were randomized to the MC group (mean [SD] age, 64.4 years [14.1]; 57 [37.5%] female) and 152 to the PICC control group (mean [SD] age, 64.8 years [12.9]; 73 [48.0%] female). All patients were followed up until 90 days after catheter removal (Figure 1). Patient, catheter, and hospital characteristics are shown in Table 1.

**Table 1.** Patient, Catheter, and Hospital Characteristics

Characteristics	Patients, No. (%)	
	MC group (n = 152)	PICC group (n = 152)
Patient age, mean (SD), y	64.4 (14.1)	64.8 (12.9)
Sex		
Female	57 (37.5)	73 (48.0)
Male	95 (62.5)	79 (52.0)
Body mass index, mean (SD)^a^	28.1 (5.9)	27.7 (7.0)
Indication		
Antibiotics	65 (42.8)	63 (41.4)
Fluids	6 (3.9)	3 (2.0)
Antibiotics and fluids	59 (38.8)	62 (40.8)
Difficult intravenous access	18 (11.8)	12 (7.9)
Other (ie, chemotherapy)	4 (2.6)	12 (7.9)
Charlson Comorbidity Index score, median (IQR)	2 (1-4)	1 (0-3)
Charlson Comorbidity Index elements		
Myocardial infarction	21 (13.8)	13 (8.6)
Congestive heart failure	14 (9.2)	13 (8.6)
Peripheral vascular disease	34 (22.4)	19 (12.5)
Cerebrovascular disease	12 (7.9)	11 (7.2)
Dementia	0	0
Chronic pulmonary disease	37 (24.3)	39 (25.7)
Connective tissue disease	11 (7.2)	9 (5.9)
Ulcer disease	0	3 (2.0)
Mild liver disease	2 (1.3)	2 (1.3)
Moderate to severe liver disease	3 (2.0)	3 (2.0)
Diabetes	13 (8.6)	20 (13.2)
Diabetes with end organ damage	28 (18.4)	19 (12.5)
Hemiplegia	5 (3.3)	2 (1.3)
Kidney disease	10 (6.6)	7 (4.6)
Any tumor without metastasis including leukemia and lymphoma	32 (21.1)	23 (15.1)
Metastatic solid tumor	17 (11.2)	13 (8.6)
AIDS	0	0
HIV-related cancer	0	0
Time spent on insertion, median (IQR), min	10.0 (7.3-12.0)	11.0 (10.0-15.0)
Insertion attempts		
1	128 (84.2)	115 (75.7)
≥2	24 (15.8)	37 (24.3)
Accidental arterial puncture	1 (0.7)	1 (0.7)
Length of catheter, median (IQR), cm	15 (15-15)	44 (41-46)
Catheter size, French		
4	152 (100.0)	21 (13.8)
5	0	131 (86.2)
Access vein		
Cephalic	4 (2.6)	4 (2.6)
Basilic	128 (84.2)	139 (91.5)
Brachial	20 (13.2)	9 (5.9)
Tip placement on radiograph		
Subclavian vein	NA	5 (3.3)
Superior vena cava	NA	112 (73.7)
Right atrium	NA	28 (18.4)
Other	NA	3 (2.0)
Missing	NA	4 (2.6)
Dwell time, median (IQR), d	10 (6-15)	11 (6-17)
Readmission within 90 d	60 (39.5)	49 (32.2)
90-d mortality	14 (9.2)	15 (9.9)

Abbreviations: MC, midline catheter; NA, not applicable; PICC, peripherally inserted central catheter.

^a^ Body mass index is calculated as weight in kilograms divided by height in meters squared.

**Figure 2.**
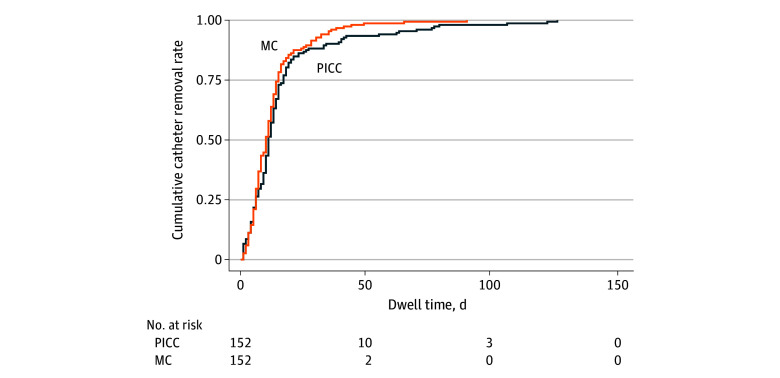
Unadjusted Kaplan-Meier Estimates of Cumulative Catheter Removal Rates MC indicates midline catheter; PICC, peripherally inserted central catheter.

In the MC group, there were no patients with a CRBSI, whereas a single CRBSI was observed in the PICC control group (*P* > .99). Symptomatic DVT occurred in none of the patients in the MC group and in 2 patients in the PICC control group, leading to a premature catheter removal in 1 of these patients (*P* = .50). A total of 274 patients (90.1%) had a functional catheter until end of treatment. However, premature catheter removal occurred in 20 patients (13.2%) with an MC compared with 10 patients (6.6%) with a PICC (*P* = .045). The incidence of premature catheter removal was 10.3 and 3.9 removals per 1000 catheter-days for the MC group and PICC control group, respectively (*P* = .02). The primary reasons for removal are shown in Table 2. Patients who received MCs had 2.61 times higher incidence rate for premature catheter removal compared with those who received PICCs (IRR, 2.61; 95% CI, 1.21-5.64; *P* = .02). Additionally, 1 patient in the PICC group had a complication (DVT) without premature catheter removal. The complication rate was 13.2% (20 patients) in the MC group compared with 7.2% (11 patients) in the PICC group, and the incidence rate for catheter complications was 2.37 times higher among patients with a MC compared with those with a PICC (IRR, 2.37; 95% CI, 1.12-5.02; *P* = .02). Of the complications observed, only 3 (9.7%) were categorized as major, involving CRBSI and DVT, while the remaining 28 (90.3%) were classified as minor (Table 3). Unadjusted Kaplan-Meier estimates of cumulative catheter removal are shown in Figure 2.

**Table 2.** Catheter-Related Complications

Outcome	Patients, No. (%)		*P* value
	MC group (n = 152)	PICC group (n = 152)	
Primary			
CRBSI	0	1 (0.7)	>.99
Secondary			
Deep vein thrombosis	0	2 (1.3)	.50
Reasons for catheter removal			
Treatment ended	132 (86.8)	142 (93.4)	.045
Pain during infusion	2 (1.3)	0	
Phlebitis	0	1 (0.7)	
Infiltration	2 (1.3)	0	
Accidental removal	4 (2.6)	1 (0.7)	
Occlusion	3 (2.0)	2 (1.3)	
Leaking	7 (4.6)	1 (0.7)	
Other	2 (1.3)	5 (3.3)	
Premature removal, all cause	20 (13.2)	10 (6.6)	.08
Incidence of premature catheter removal per 1000 catheter-days (95% CI)	10.3 (6.6-15.9)	3.9 (2.1-7.3)	.02

Abbreviations: CRBSI, catheter-related bloodstream infection; MC, midline catheter; PICC, peripherally inserted central catheter.

**Table 3.** Complications and Premature Catheter Removal

Complication	Patients, No. (%)		IRR (95% CI)	*P* value
	MC group (n = 152)	PICC group (n = 152)		
Any complications	20 (13.2)	11 (7.2)	2.37 (1.12-5.02)	.02
Premature removal	20 (13.2)	10 (6.6)	2.61 (1.21-5.64)	.02
Major complications^a^	0 (0.0)	3 (2.0)	NA	NA
Minor complications^b^	20 (13.2)	8 (5.3)	3.26 (1.40-7.59)	.006

Abbreviations: IRR, incidence rate ratio; MC, midline catheter; NA, not applicable; PICC, peripherally inserted central catheter.

^a^ Major complications included catheter-related bloodstream infection and deep vein thrombosis.

^b^ Minor complications included pain during infusion, phlebitis, infiltration, accidental removal, occlusion, leaking, and other reasons.

In a subgroup analysis, we stratified by catheter dwell time, distinguishing between catheters used for less than 16 days and those used for 16 days or more. For catheters with a dwell time of less than 16 days, there was no difference in any complications (IRR, 1.16; 95% CI, 0.50-2.68; *P* = .73) or premature catheter removals (IRR, 1.29; 95% CI, 0.54-3.06; *P* = .57) between the catheter groups. However, among catheters with a dwell time of 16 days or more, patients in the MC group had more complications and premature removals (IRR, 13.18; 95% CI, 1.75-99.39; *P* = .01) compared with patients in the PICC control group (data not shown).

## Discussion

To our knowledge, this is the largest RCT comparing the safety and efficacy of a new generation MCs with PICCs. We found only 1 CRBSI, with no difference between the catheter groups. However, we observed an increase in all-cause catheter complications in the MC group when compared with the PICC control group, with an IRR of 2.37. This observed difference was primarily driven by an increased incidence of complication within the MC group when catheters were used for more than 15 days compared with the PICC control group.

Our findings are consistent with a meta-analysis from 2021,^14^ which found a risk of developing a CRBSI at 0.58% in MCs and 0.48% in PICCs. A large cohort study from 2022 examined the efficacy of MCs compared with PICCs in more than 10 000 patients and found a prevalence of CRBSI at 0.4% for the MC group and 1.6% for the PICC group.^15^ Both studies support the low CRBSI findings in present study. The overall incidence of catheters removed due to infection in the present study was negligible, with only 1 superficial infection besides the single CRBSI found, which may be explained by the sterile technique used in the hands of skilled nurses. Another single-center Danish RCT^16^ found that MCs can be inserted successfully at bedside with a nontouch technique in the hands of a single operator. However, in this study the reported infection rate was 18%, all leading to catheter removals. This highlights the importance of rigorous hygiene to secure catheter patency from insertion until completion of therapy.

We found that 13.2% of patients with MCs and 7.2% of patients with PICCs had catheter-related complications. The absolute incidence of MC complications was low compared with other findings in the literature, with rates ranging from 15% to as high as 38%.^16,17,18^ In a recent systematic review, where all different types of MCs in different lengths were pooled in the analysis, the all-cause complication rate was 12.5% (95% CI, 11.9%-13.2%).^19^ This review primarily consisted of cohort studies, with only a few smaller RCTs included, and not all studies had a comparator. As a result, not all types of complications were consistently reported, leading to potential underreporting of all-cause complication rates. Another study found that when the catheter-to-vein ratio exceeds 45%, the risk of complications increases.^20^ In our insertion procedures, we did not routinely assess vein diameter or adapt catheter thickness accordingly. This highlights the need to select the right catheter for the patient, considering the anticipated treatment duration and type of IV therapy to avoid multiple insertions and secure catheter patency throughout the entire treatment period.

In the PICC control group, catheter tip placement was routinely verified with postprocedure radiographs. However, this practice was not applied to the MC group. While the use of ultrasonography guidance to optimize the MC tip location has been demonstrated as feasible, its effect on reducing complications remains uncertain.^21^

Familiarity with use of MCs and PICCs might also be important. In a feasibility study involving a pediatric population, introduction of a new MC was compared with a PICC-based algorithm.^22^ In this study, 19% of the clinicians had used the MCs fewer than 6 times prior to study initiation. Consequently, more complications with use of MCs were observed at the beginning of the study, which highlights the potential impact of a varied learning curve when introducing a new device into routine health care. To address this issue, we ensured that the vascular access team had the appropriate level of experience before the study initiation. This proactive approach may have contributed to the low rate of complications observed with both catheters in this study. By prioritizing comprehensive training and expertise in the vascular access team and among the nurses on the wards and in the home care institutions, we sought to create a foundation for successful catheter use, thereby optimizing patient safety. Continuous education and ongoing support for health care professionals are important to maintaining high standards of care and achieving optimal patient outcomes.^23^

### Limitations

Our trial had some limitations that warrant consideration. First, the external validity was low, as the study was conducted at a single site, and the catheters were placed by a few skilled vascular access nurses. Consequently, the use of MCs in other settings should be adapted, implemented, and evaluated in this context.^24^

Furthermore, patients who were assessed for eligibility and had 1 or more of the exclusion criteria or declined to participate were not registered. This study was conducted during the COVID-19 pandemic, which reduced the possibilities for registration and time spent screening patients. Therefore, there is a risk of potential selection bias, which is reduced by the randomized design of the study.

Moreover, we were unable to blind the intervention. This led to a potential risk of detection as well as performance bias. Nevertheless, the primary outcome, CRBSI, and the secondary outcomes, DVT and catheter failures of different reasons, are comparators with clear objective findings that are unlikely to be affected by the participants, vascular access nurses, nurses on the wards and in the home care institutions, or the research team’s knowledge about the intervention.

In this study, no systematic blood or catheter tip cultures were drawn, which could lead to a risk of underreporting the real incidence of the primary outcome. However, the diagnostic standards were described in advance and based on objective results, and we assumed no different diagnostic criteria according to catheter type. Additionally, the risk of asymptomatic cases of DVT might have led to some underreporting of this event, but we considered this to be independent of catheter type. Thus, the risk of misclassification may have led to a general underreporting of the outcomes in both groups, introducing bias toward the null.

## Conclusions

This RCT found no significant difference in CRBSI between MCs and PICCs. Both catheters were safe and demonstrated high efficacy. The incidence of catheter-related complications was higher in the MC group compared with the PICC control group. Despite this, our findings indicate that MCs could be considered an alternative to PICCs. Further cost-effectiveness studies comparing these devices are needed to balance between catheter efficacy and economic consequences.

## Notes

Supplement 1.
Trial Protocol

Supplement 2.
Data Sharing Statement
