# Supplementary material for: Safety and Efficacy of Midline vs Peripherally Inserted Central Catheters Among Adults Receiving IV Therapy: A Randomized Clinical Trial
Source: JAMA Netw Open. 2024 Feb 13;7(2):e2355716. doi: 10.1001/jamanetworkopen.2023.55716 (PMC10865154; doi:10.1001/jamanetworkopen.2023.55716)
Supplement: Supplement 1. — Trial Protocol [file jamanetwopen-e2355716-s001.pdf]

AALBORG UNIVERSITETSHOSPITAL
- i gode hænder

Comparison of two peripherally inserted catheters: a central venous and a midline

Sammenligning af to perifert anlagte katetre: et central venøst versus et midline

Protocol version 2, 3th of September 2018

Applicable protocol registration numbers:

ClinicalTrial.gov identifier: NCT04140916

1 Table of contents

2 Abstract: 4

3 Administrative information 5

4 Introduction and background 6
- 4.1 Introduction 6
- 4.2 Background 6
- 4.3 The trial 7
- 4.4 Risk and benefits by participating in the trial 7
- 4.5 Trial conduct 7
- 4.6 Schedule for study conduct including time line for key study milestones 8

5 Trial objective and purpose 8
- 5.1 Objective and purpose 8

6 Trial design 8
- 6.1 Design 8
- 6.2 Method 8
  - 6.2.1 Catheter placement 8
  - 6.2.2 Trial flow-chart 9

7 Selection of participants 9
- 7.1 Inclusion criteria 9
- 7.2 Exclusion criteria 9

8 Outcomes 10
- 8.1 Primary outcome 10
- 8.2 Secondary outcome 10

9 Recruitment procedures and data collection 10
- 9.1 Recruitment procedure 10
- 9.2 Data collection 10
  - 9.2.1 Method 10
  - 9.2.2 Variables 11

10 Ethical considerations 12
- 10.1 Ethical justification and trial rationale 12

11 Data handling and record keeping 13
- 11.1 Data management 13
- 11.2 Confidentiality 13
- 11.3 Access to data 13

12 Statistical plan and data analysis 13
- 12.1 Sample size estimation and power calculation 13
- 12.2 Statistical methods 13
  - 12.2.1 Significance 13

13 Legal and organisational aspects 14

13.1 Finance 14
13.2 Compensation 14
13.3 Insurance 14
13.4 Plan for publication, authorship and dissemination 14
13.5 Intellectual property rights..14
14 References...14

2 Abstract

Background: Central venous access via catheter insertion has become a very common practice in the hospital and outpatient settings for various purposes, including hemodynamic monitoring, infusion of irritant drugs like chemotherapy or total parenteral nutrition (TPN), poor peripheral venous access or long term administration of drugs such as antibiotics. The overall complication rate is more than 15% and a great preventive effort is done. Catheter Related Bloodstream Infection (CRBSI) and deep vein thrombosis are serious and feared complications associated with prolonged hospital stays, increased costs and risk of mortality**.**
Peripherally inserted central catheter (PICC) is a central venous catheter (CVC) which is easy to place, safe and cost-effective and a well-established alternative compared with other CVCs.
Midline is another peripherally inserted catheter, which by definition is 7.5 to 20 centimetres long (3-8 inches) and thus not a central venous catheter. It is inserted in the same peripherally veins as the PICC, but the tip is advanced no further than the distal axillary vein. The midline cannot be used to vesicants or irritants like most chemotherapy, vasoactive agents, TPN or medications with extremely low or high pH values. The midline is suitable for use from 5 days until 4 weeks.

Objectives: To assess the efficacy and safety of midline catheters using standard care with PICC as reference.

Design: Single-center non-blinded randomised clinical trial.

Inclusion and exclusion criteria: We will assess eligibility among all patients to whom staff from a general ward requests a CVC and who meet the inclusion criteria: 1) over the age of 18 years, 2) indication for intravenous medicine or fluids for 5 to 28 days and 4) have given informed consent. We will exclude patients fulfilling one or more of the exclusion criteria: 1) have infection or burns at both upper extremities, 2) are pregnant, 3) have a CVC already in place or 4) earlier randomized to the study.

Methods: With the patient supine ultrasound is used to identify the desired vein on the relevant upper extremity with full sterile coverage and after chlorhexidine preparation a catheter is placed using the Seldinger technique. Successful placement in a vein is secured by aspirating blood from the catheter. Depending on the randomization a PICC is placed and the tip position in vena cava superior is verified by a chest x-ray or a midline is placed without the need of x-ray verification.

Outcomes: Primary outcome: Any registered CRBSI in the time window from placement to removal of the catheter, defined by clinical signs of infection and at least one positive blood culture in the absence of other apparent source for the infection, except the catheter. In addition, a quantitative catheter tip culture with the same organism isolated from the catheter segment and peripheral blood culture also defines a CRBSI. Secondary outcome: Any registered other complication in the time window from placement to removal of the catheter, including DVT, catheter failure of mechanical cause, phlebitis, infiltration, pain in relation to drug or fluid administration or leaking of blood or fluids from the puncture site.

Trial size: Based on an expected incidence of CRBSI at 5% in the PICC group with reference to the literature and an expected incidence of 0% in the midline group with reference to a follow-up of the first 107 midline catheters inserted in patients at Aalborg University Hospital, an alpha of 0.05 and a beta of 0.2 (power 0.8) the sample size is 304 with 152 patients in each group.

3 Administrative information

This trial is initiated by Simon Ladehoff Thomsen.
The Department of Anaesthesia and Intensive Care, Aalborg University Hospital finances all costs associated with the trail.

Clinical responsible
Simon Ladehoff Thomsen, MD,
Telephone: 97 66 18 64

Responsible investigator
Professor Bodil Steen Rasmussen, MD, PhD
Telephone: 97 66 18 93

Project group
Professor Bodil Steen Rasmussen, MD, PhD [1)]
Simon Ladehoff Thomsen, MD[1)]
Rikke Boa, nurse anesthetist[1)]
Anita Olsson, nurse anesthetist[1)]
Marianne Levin, Critical care nurse[1)]
Lars Winther Jensen, MD [2)]
1) Department of Anaesthesia and Intensive Care, Aalborg University Hospital
2) Centre for Nutrition and Bowel Disease, Department of Gastroenterology, Aalborg University Hospital

Trial site
Department of Anaesthesia and Intensive Care, Aalborg University Hospital

4 Introduction and background

4.1 Introduction

In the United States more than 5 million patients each year get a central venous catheter[1]. Indications for central venous catheterization include infusion of irritant drugs like chemotherapy or total parenteral nutrition (TPN), poor peripheral venous access and long term administration of drugs such as antibiotics[2]. This ubiquitous procedure has many associated complications that result in morbidity, mortality, and increased healthcare cost. The overall complication rate is more than 15% and a great preventive effort is done[1].

4.2 Background

Catheter Related Bloodstream Infection (CRBSI) is a serious and feared complication associated with prolonged hospital stays, increased costs and risk of mortality[3]. CRBSI is defined as the presence of bacteremia originating from an intravenous catheter. A lot of effort is done to reduce CRBSI including heightened attention to hygiene in placement and care, improved education and training, and placement of a team with specialized skills. Adherence to best practice for central line placement is shown to reduce risk of CRBSI[4].

Central venous catheters (CVCs) are associated with deep vein thrombosis (DVT) and pulmonary embolism[5]. Besides interruption in treatment, catheter-related DVT increases morbidity and mortality. Cancer and admission to intensive care are independent risk factors. Existing data report wide estimates of this adverse outcome, ranging from less than 1% to as high as 38.5%, dependent on the population studied, method of diagnosis, and use of prophylaxis measures[5].

Peripherally inserted central catheter (PICC) is a CVC which placement and use have been widespread since it was first described in 1975[6]. It is a well-established alternative to CVCs placed via for example the subclavian or jugular veins. It is easy to place, safe and cost-effective compared to others often used central lines. PICC is inserted via a peripheral vein in the upper arm and terminates like other central lines in the vena cava superior. Placement and use is associated with few complications[7].

Another peripherally inserted catheter is the midline which by definition is 7.5 to 20 centimeters long (3-8 inches) and thus not a central venous catheter. The midline catheter was introduced in 1950s[8]. It has since that time undergone major improvement in material technology and techniques for achieving vascular access. It is inserted in the same peripherally veins as the PICC, but the tip is advanced no further than the distal axillary vein and is therefore classified as a peripheral intravenous catheter with corresponding advantages and disadvantages. The midline cannot be used to vesicants or irritants like most chemotherapy, vasoactive agents, TPN or medications with extremely low or high pH values. The midline is suitable for use from 5 days until 4 weeks to drugs and solutions, which safely can be administrated in a peripheral venous catheter. Severe complication to placement and use of midline is rare, but due to previous problems primarily related to the midline catheter material, its use is limited [9].

In a large review from 2006 the incidence of CRBSI among in- and outpatients having PICC or midline were estimated to 3.1% (95% CI 2.6-3.7) and 0.4% (95% CI 0.0-0.9), respectively[10]. It has been demonstrated that central line use can be decreased through the use of midline catheters[11].

A retrospective descriptive review from two hospitals in America showed the effectiveness of implementing a midline program resulted in a 78% reduction in central line-associated bloodstream infection[12]. In a similar Australian retrospective cohort study in a ventilator unit population, a significant decrease in the rate of central line-associated bloodstream infections was found after use of midlines in place of central lines[13]. It seems that the introduction and regular use of midlines when warranted may reduce the overall incidence of CRBSI and its sequelae in certain hospital environments.

In a meta-analysis including 11,476 hospital admitted patients with PICC, DVT was found in 3.44% (95% CI 2.46-4.43)[5]. DVT occurrence in relation to midline is understudied, but is reported with a low incidence between 0-2 %[14,15,16]. The overall incidence of DVT and related potentially secondary complications of both catheters seems low. The risk of minor complications such as pain, leakage or phlebitis is in a retrospective comparison study found to be 11.5% for midlines and 1.5% for PICC, respectively (P<0.001)[13].
The efficacy of the PICC is well studied, the incidence of side effects is known and its use is implemented all over the world. To our knowledge, no-one has compared the efficacy of midlines with a CVC in a prospective study. In this study we will examine the efficacy and safety of midline catheters using standard care with PICC as reference.

4.3 The trial

Placement and use of PICC has since 2008 been well-established in our department and is standard care to patients who needs TPN, long term intravenous therapy or chemotherapy.
The applicability and overall complications of the midline catheter is understudied and mostly based on retrospective data. Midline has the potential to optimize the treatment of a wide range of patient categories as the material has been approved significantly. Since September 2017 we have tested the midline catheter, and after over 100 placements, we use it with success as an integrated offer in our intravenous access team.
Patients eligible for screening for inclusion are identified among all patients where staff from a general ward requests a central line. In the Department of Anaesthesia upon the primary contact, the anesthesiologist in charge or special trained nurse will perform the inclusion process and randomization. The randomization is 1:1 between the PICC (control group) and a midline catheter (intervention group). After placement the patients will be closely followed until the day of removal of the catheter. To obtain information on length of hospital stay and mortality, the electronical journal will be checked until 90 days after catheter removal. The Incidence of complications that occur will be registered and the two catheter groups will be compared.

4.4 Risk and benefits by participating in the trial

In patients with poor peripheral venous access or need of long time administration of medicine or fluids a central line is routinely placed. In our department it is standard care to place a PICC in these patients. The puncture site on the upper arm and placement technique are the same for both catheter types. Therefore the complications during placement are expected to be independent of catheter type. PICC require x-ray confirmation of tip placement, leading to additional costs and exposing the patient to unnecessary radiation. X-ray verification of tip position is not necessary when placing the midline catheter. By participating in this study the patients have benefit of more close observation and patients in the intervention group have the possible benefit of reduced risk of having a CRBSI. In the intervention group, the risk of minor complications like pain in relation to fluid or medicine administration, infiltration or phlebitis is expected to be increased compared with the control group. The discomfort in relation to the minor complications is expected to disappear without any persistent complications.

4.5 Trial conduct

The trial will be conducted in accordance with the published study protocol, under the principles of the Helsinki declaration and after approval by the local committee on health research ethics and the Danish

Data Protection Agency according to Danish law. The protocol will be registered on www.clinicaltrials.gov before the trial is started.

4.6 Schedule for study conduct including time line for key study milestones

- Start of preparation: 1st of August 2018
- Start of clinical trial: 1st of September 2018
- End of clinical trial: 1st of March 2020
- End of data processing and analysis: 1th of June 2020

5 Trial objective and purpose

5.1 Objective and purpose

By registering severe and minor complications to intravenous catheters we will show the difference between the PICC, which is standard care, and the midline catheter among in- and out-hospital patients for whom the ward requests a central venous access.

6 Trial design

6.1 Design

Single-center randomised controlled trial with in- and out-patients from medical and surgical departments on Aalborg University Hospital, Denmark.

6.2 Method

6.2.1 Catheter placement

The informed consent and randomization is performed in the Department of Anaesthesia prior to placement of catheter described below in section 9.1.
With the patient supine ultrasound is used to identify the desired vein on the relevant upper extremity. Full sterile technique is used and includes the operator wearing sterile gown, mask, cap, and sterile gloves. The area is then prepared with chlorhexidine followed by adequate sterile draping. The Seldinger technique is used to insert the catheter. Successful placement in a vein is secured by aspirating blood from the catheter. The catheter is then flushed with minimum 20 mL saline.
Depending on the randomization a PICC is placed and the tip position in vena cava superior is verified by a chest x-ray or a midline is placed without the need of x-ray verification. The patient is then returned to the medical or surgical ward.

6.2.2 Trial flow-chart

The following consort diagram will be continuously filled out to monitor study progress.

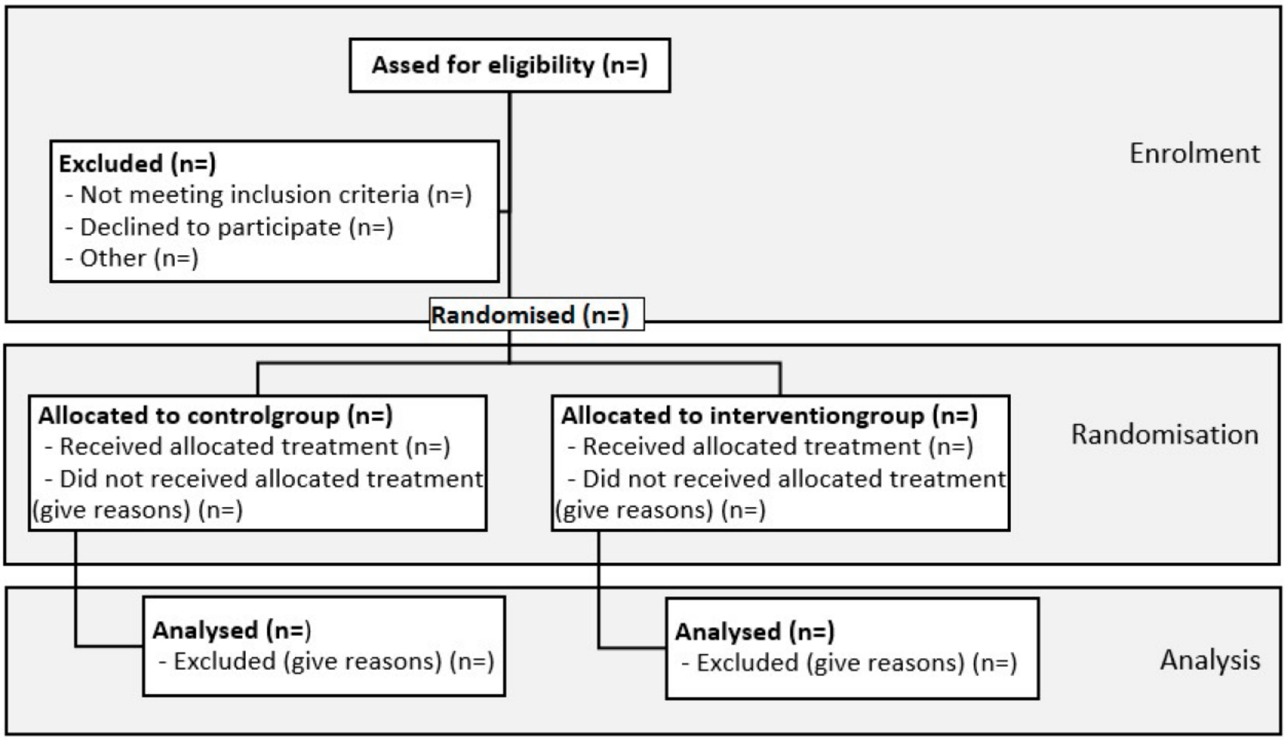

7 Selection of participants

7.1 Inclusion criteria

- Over the age of 18 years *(The age of the patient in whole years at the time of the trial. The age will be calculated from date of birth)*
- Indication for intravenous medicine or fluids included in the following:
    - Blood products, isotonic saline- or glucose-solutions (including glucose-insulin-potassium-solutions)
    - Antibiotics (penicillins, cephalosporins, carbapenems or fluoroquinolones)
    - Chemotherapy registered for use in a peripheral vein catheter
- Expected indication for intravenous access in 5 to 28 days (*Evaluated by ward staff*)
- Informed consent *(Defined in 5.1)*

7.2 Exclusion criteria

- Infection or burns at both upper extremities (*Involving the area of puncture site*)
- Pregnancy *(Confirmed by positive urine human gonadotropin (hCG) or plasma-hCG)*
- A central venous catheter already in place *(Self-explanatory)*
- Earlier randomized to the study *(Self-explanatory)*

8 Outcomes

8.1 Primary outcome

Any registered CRBSI in the time window from placement to removal of the catheter *(Defined by clinical signs of infection (i.e., fever, chills, leukocytosis or hypotension) and at least one positive blood culture obtained from a peripheral vein and/or from venous access devise in the absence of other apparent source for the infection, except the catheter. In addition, a quantitative (>1000 colony-forming units /catheter segment) catheter tip culture with the same organism (species and anti-biogram) isolated from the catheter segment and peripheral blood culture also defines a CRBSI)*

8.2 Secondary outcome

Any registered other complications in the time window from placement to removal of the catheter:

- DVT (*Defined by the formation of one or more symptomatic or non-symptomatic blood clots in a large vein verified by ultrasound (US) or computed tomography (CT) scan*)
- Catheters failure of mechanical cause (*Fallen out, pulled out by mistake, occluded, broken or other defects*)
- Phlebitis defined as 2 or more on the phlebitis scale (*see below*)
- Infiltration defined as 2 or more on the infiltration scale (*see below*)
- Pain in relation to drug or fluid administration (*Defined as above 3 cm on the Pain Visual Analog Scale*)
- Leaking of blood or fluids from the puncture site (*Evaluated by the ward staff*)

9 Recruitment procedures and data collection

9.1 Recruitment procedure

Subjects are recruited at Aalborg University Hospital among patients where staff from a general ward requests a central line. The anesthesiologist or special trained nurse responsible for inclusion will apply to the national regulations regarding informed consent to participation in a clinical trial. Hence, in addition to oral information the potential participant will receive written information including both the specific information on the current study as well as the general information pamphlet on participant rights when entering a clinical trial. All information and inclusion will be given by physicians or special trained nurses who possess the sufficient professional prerequisites to be authorized by the sponsor to have a direct involvement in the project.
Information will be given in private and the participant will be allowed to have an assessor present. The participant will be given a brief reflection period before making their decision. As always it is voluntary to participate and the subjects can withdraw their commitment to participate at any time.

9.2 Data collection

9.2.1 Method

Participants will be randomized in either the intervention or the control group using the online randomization tool Research Electronic Data Capture (REDCap), Aarhus University, Denmark.
All baseline information and data from the placement procedure in the Department of Anaesthesia will be registered on paper and directly entered into the REDcap database. A registration paper will follow the patient to the ward and the staff will daily register the occurrence of the primary or secondary

endpoints. All registration sheets will subsequently be collected by the investigators and entered online in the Redcap database. Participants agree with their participation that investigators will use their electronic journal. The electronical journal will be followed to register complication not noted on the registration paper. If the patient is discharged the electronic journal will be followed for 90 days to registrar potential re-admissions and or mortality. Access for data registration and access to already submitted data will be granted to all the initiating investigators.
Information from the examination done at the admission to hospital and listed in the medical journal is used to fill out the baseline variables: age, sex, height, weight, medical history and clinical examination to make it possible to describe the cohort. For definitions see below.

9.2.2 Variables

Baseline variables:

- Date of birth *(Self-explanatory)*
- Age *(Defined in inclusion criteria)*
- Sex *(The genotypic sex of the participant)*
- Ethnicity *(Defined as race and thereby emphasizing shared physical appearance based on genetic origin)*
- Height *(In centimetres, if lower extremities are bilaterally amputated, the estimated original height should be used)*
- Weight *(In kilograms with one decimal)*
- BMI *(Weight divided with height (in meters) squared. A result between 18.5 and 25 is considered normal, less than 18.5 as underweight and more than 25 as overweight)*
- Medical history *(An account of any symptoms/illness experienced now or before by the participants)* and clinical examination *(The process by which a medical doctor investigates the body of a participant for signs of disease)* to determine Charlson Comorbidity Index.

During placement on the anaesthesia department:

- Date of randomization *(The date the individual patient have their randomization)*
- Date of placement *(If different from date of randomization)*
- Time spent on placement (*Time in minutes and seconds from first skin puncture to placement of dressing*)
- Number of skin puncture *(Self-explanatory)*
- Type and length of placed catheter (*Product manufacturer, catheter type and length in centimetres)*
- Name of the access-vein *(Name of the vein on the upper-arm)*
- Accidental arterial puncture *(Self-explanatory)*
- Bleeding complications (*arterial or venous haemorrhage during placement including haematoma or arterial aneurism*)
- Tip placement on chest x-ray (*control group only - assessed from an anterior and posterior X-ray of thorax*)

After placement on the ward:

- CRBSI (*As defined under 5.1*)
- Catheter removal date (*The date of catheter removal*)
- Catheters removal cause:

  - Pain in relation to drug or fluid administration *(Patient reported pain above 3 cm on the Pain Visual Analog Scale in relation to drug or fluid administration)*
  - Phlebitis scale score above 1:
    - *0 No symptoms*
    - *1 Erythema at access site with or without pain*
    - *2 Pain at access site with erythema or edema*
    - *3 Pain at access site with erythema or edema; streak formation; palpable venous cord*
    - 4 *Pain at access site with erythema or edema; streak formation; palpable venous cord < 2.5 cm in length; purulent drainage*

  - Infiltration scale score above 1:
    - *0 No symptoms*
    - *1 Skin blanched; edema < 2.5 cm in any direction; cool to touch; with or without pain*
    - *2 Skin blanched; edema in 2.5 to 15 cm in any directions; cool to touch; with or without pain*
    - *3 Skin blanched, translucent; gross edema > 15 cm in any directions; cool to touch; mil-to-moderate pain; possible mumbness*
    - *4 Skin blanched, translucent; skin tight; leaking: skin discoloured: bruised; swollen; gross edema > 15 cm in any direction; deep pitting tissue edema; circulatory impairment: moderate-to-severe pain; infiltration of any amount of blood product, irritant, or vesicant*

  - Mechanical cause (Fallen out, pulled out by mistake, occluded, broken or other defects)
  - Leaking of blood or fluids from the puncture site (Evaluated by the ward staff)

- Deep vein thrombosis (Verified on CT or US with or without symptoms)
- Date of death *(Self-explanatory)*
- Date of discharge *(The day the patient is discharged from hospital)*
- Date of re-admission (*The day the patient is re-admitted to hospital)*

10 Ethical considerations

10.1 Ethical justification and trial rationale

In this study we will describe the safety and efficacy of the midline catheter compared with the PICC. Regardless of a patient being treated within the study or declines participation, a central venous access

is needed. As both the midline and PICC are well established methods to secure venous access, no significant disadvantages or ethical concerns are found with conduction of the study.
If our hypothesis is confirmed, the risk of CRBSI will be reduced against acceptable increased disadvantages. The reduced number of major complications can lead to a potential minor decreased mortality in the intervention group.
It is voluntarily to participate in the trial and based on an informed and signed consent. At any moment this consent can be withdrawn by the participant. The participants will be given oral and written information about the trial before they can give their consent and before inclusion.

11 Data handling and record keeping

11.1 Data management

Data recorded during the study is stored in electronic as well as in the written form. The Danish Data Protection Agency's standard terms regarding security will be followed.

11.2 Confidentiality

Each participant will receive a unique trial identification number and all personalized information will be anonymized. Data is handled according to Danish law.

11.3 Access to data

Specially authorized persons from the local committee on health research ethics, the Danish Medicines Agency and the Danish Data Protection Agency will have unimpeded access to monitor and inspect all data and documents during the trial.

12 Statistical plan and data analysis

12.1 Sample size estimation and power calculation

The primary outcome is CRBSI. The power calculation is based on an expected incidence of 5% in the PICC group with reference to the literature and an expected incidence of 0% in the midline group with reference to a follow-up of the first 107 midline catheters inserted in patients at Aalborg University Hospital from the 5th of October 2017 to the 26th of February 2018. With an alpha of 0.05 and a beta of 0.2 (power 0.8) the sample size is 304 with 152 patients in each group.

12.2 Statistical methods

Descriptive data will be presented in a baseline table according to catheter type. For normally distributed measurements the differences between groups will be compared using Student´s t-test. Variables considered not to be normally distributed will be analysed by Mann-Whitney´s U-test.
The results from primary and secondary endpoints will be presented in a separate table also according to catheter type. The differences between groups will be compared using Wilcoxon two-samples test/Fisher´s exact or unpaired t-test. Statistical analyses will be performed using STATA software (version 14; STATA, Corporation, College Station, TX)

12.2.1 Significance

A two-sided P value of less than 0.05 will be considered statistical significant.

13 Legal and organisational aspects

13.1 Finance

All initiative for the current study has been taken by the previously stated members of the project group. They have no personal economic involvement, no possible economic gain from any outcome and no ties to the medical industry or corporations gaining from study outcomes. Funding for the purchase of iPads for data registration and statistical assistance in the analysis phase will be sought from relevant private and public funds. Due to the modest running expenditures comprising only the catheters already present at the hospital, the study will be conducted regardless of any successful funds applications.

13.2 Compensation

No benefits are paid to the patients for participating in the study.

13.3 Insurance

The participants are covered according to Danish law.

13.4 Plan for publication, authorship and dissemination

All positive, negative and inconclusive results are published via www.clinicaltrials.gov.
Our plan is to write at least one paper for publication.
"Comparison of peripherally inserted central catheters and midlines; a randomized and controlled trial", S.L. Thomsen, R. Boa, A. Olsson, M. Levin L. W. Jensen, B. S. Rasmussen, journal, xx 2020.
Results are presented at the annually meeting of the Danish Society of Anaesthesiology and Intensive care Medicine or at an international conference.
No data can be retained from publication.

13.5 Intellectual property rights

As according to general guidelines.

14 References

---

[1] McGee DC, Gould MK. Preventing complications of central venous catheterization. N Engl J Med. 2003 Mar 20;348(12):1123-33.

[2] Smith RN, Nolan JP. Central venous catheters. BMJ. 2013 Nov 11;347:f6570.

[3] Gahlot R, Nigam C, Kumar V, Yadav G, Anupurba S. Catheter-related bloodstream infections. Int J Crit Illn Inj Sci. 2014 Apr;4(2):162-7.

[4] Centers for Disease Control and Prevention (CDC). Vital signs: central line-associated blood stream infections--United States, 2001, 2008, and 2009. MMWR Morb Mortal Wkly Rep. 2011 Mar 4;60(8):243-8.

[5] Chopra V, Anand S, Hickner A, Buist M, Rogers MA, Saint S, Flanders SA. Risk of venous thromboembolism associated with peripherally inserted central catheters: a systematic review and meta-analysis. Lancet. 2013 Jul 27;382(9889):311-25.

[6] Hoshal VL. Total intravenous nutrition with peripherally inserted silicone elastomer central venous catheters. Arch Surg. 1975 May;110(5):644-6.

[7] Chopra V, Anand S, Krein SL, Chenoweth C, Saint S. Bloodstream infection, venous thrombosis, and peripherally inserted central catheters: reappraising the evidence. Am J Med. 2012 Aug;125(8):733-41.

[8] Anderson NR. Midline catheters: the middle ground of intravenous therapy administration. J Infus Nurs. 2004 Sep-Oct;27(5):313-21.

[9] Adams DZ[1], Little A[2], Vinsant C[1], Khandelwal S[1]. The Midline Catheter: A Clinical Review. J Emerg Med. 2016 Sep;51(3):252-8.

[10] Maki DG, Kluger DM, Crnich CJ. The risk of bloodstream infection in adults with different intravascular devices: a systematic review of 200 published prospective studies. Mayo Clin Proc. 2006;81:1159–71.
[11] Pathak R, Patel A, Enuh H, Adekunle O, Shrisgantharajah V, Diaz K. The Incidence of Central Line-Associated Bacteremia After the Introduction of Midline Catheters in a Ventilator Unit Population. Infect Dis Clin Pract (Baltim Md). 2015 May;23(3):131-134.
[12] Moureau N, Gordon S, Margaret H. How to Establish an Effective Midline Program: A Case Study of 2 Hospitals. J Vasc Access. 2015 Sept; 20(3):179-188
[13]Pathak R, Patel A, Enuh H, Adekunle O, Shrisgantharajah V, Diaz K. The Incidence of Central Line-Associated Bacteremia After the Introduction of Midline Catheters in a Ventilator Unit Population. Infect Dis Clin Pract (Baltim Md). 2015 May;23(3):131-134
[14] Xu T, Kingsley L, DiNucci S, Messer G, Jeong JH, Morgan B, Shutt K, Yassin MH. Safety and utilization of peripherally inserted central catheters versus midline catheters at a large academic medical center. Am J Infect Control. 2016 Dec 1;44(12):1458-1461.
[15] Caparas JV, Hu JP. Safe administration of vancomycin through a novel midline catheter: a randomized, prospective clinical trial. J Vasc Access. 2014 Jul-Aug;15(4):251-6.
[16] Dumont C1, Getz O, Miller S. Evaluation of midline vascular access: a descriptive study. Nursing. 2014 Oct;44(10):60-6.
