# Supplementary material for: Safety and Efficacy of Midline vs Peripherally Inserted Central Catheters Among Adults Receiving IV Therapy: A Randomized Clinical Trial
Source: JAMA Netw Open. 2024 Feb 13;7(2):e2355716. doi: 10.1001/jamanetworkopen.2023.55716 (PMC10865154; doi:10.1001/jamanetworkopen.2023.55716)
Supplement: Supplement 2. — Data Sharing Statement [file jamanetwopen-e2355716-s002.pdf]

Data Sharing Statement

Thomsen. Safety and Efficacy of Midline vs Peripherally Inserted Central Catheters Among Adults Receiving IV Therapy. *JAMA Netw Open*. Published February 13, 2024. doi:10.1001/jamanetworkopen.2023.55716

Data
Data available: No
